# Redefining the boundary: a rationale for lowering the upper age limit for early childhood caries to 60 months

**DOI:** 10.3389/froh.2026.1783470

**Published:** 2026-03-18

**Authors:** Moréniké Oluwátóyìn Foláyan

**Affiliations:** Department of Child Dental Health, Obafemi Awolowo University, Ile-Ife, Nigeria

**Keywords:** age factors, child, deciduous, dental caries, epidemiologic studies, health policy, keyword, pediatric dentistry

## Introduction

For decades, the dental community has utilized the standardized definition of Early Childhood Caries (ECC), established by a pivotal 1999 consensus workshop, as the presence of one or more decayed, missing (due to caries), or filled tooth surfaces in any primary tooth in a child under 71 months (1). This definition, reaffirmed by major bodies like the American Academy of Pediatric Dentistry and the International Association of Paediatric Dentistry (2), was a direct response to profound terminological chaos. It replaced pejorative labels like “nursing caries” that implied a narrow aetiology, stigmatized parents, and failed to capture the disease's multifactorial nature (3). By introducing a common, neutral terminology, the workshop provided a crucial epidemiological tool, enabling valid comparisons of prevalence data and catalysing a coherent global effort to understand the disease burden (1).

The success of this standardization now provides the very foundation for a more nuanced definition. While the current age cutoff of 71 months was a pragmatic consensus for its time, subsequent advancements in our understanding of dental development, disease progression, and integrated health systems reveal critical limitations. This manuscript is an argumentative review that selectively examines and synthesizes existing literature, not to provide a comprehensive summary of all evidence on ECC, but to build a focused, persuasive case supporting a specific position: the redefinition of ECC by lowering its upper age limit from 71 months to 60 months to better reflect contemporary biological realities, enhance precision in public health, and foster seamless integration with general child healthcare. The narrative is structured to present a logical rationale, using cited evidence to affirm the argument's validity while acknowledging and integrating counterpoints to strengthen the overall argument.

## The biological imperative: aligning with contemporary dental development

The most compelling argument for change lies in the realm of dental development. The mixed dentition phase, marked by the eruption of the first permanent molars and incisors, has traditionally been considered to begin around six years of age (4). This seemingly informed the upper cut-off age for ECC, thereby confining the disease to the primary dentition. However, a growing body of evidence confirms a trend toward earlier dental maturation. Significant proportions of children now experience the eruption of their first permanent teeth, typically the mandibular central incisors or first molars, at age five or younger (5).

A systematic review and meta-analysis reveal that the first permanent mandibular molars often erupt before the age of 6 years. This trend is most pronounced in African populations, where the right first molar erupts at a mean age of 5.40 years (95% CI: 4.85–5.95). In Asian and European populations, eruption may also occur before 6 years in some individuals, as indicated by the lower bounds of the confidence intervals (5.92 years for both regions). Similarly, permanent central incisors may erupt before 6 years in African populations (lower CI bound: 5.65 years), whereas in Asian and European groups, central incisor eruption typically occurs after this age (5). These findings demonstrate that the traditional eruption timeline no longer accurately represents contemporary paediatric populations. This shift, likely influenced by genetic (6), environmental (7), and nutritional (8) factors, means that many children now enter the mixed dentition phase before reaching the current age limit used to define ECC.

Categorizing caries in a newly erupted permanent tooth as ECC is physiologically inconsistent, as it conflates two biologically distinct stages of dentition with differing susceptibility, risk profiles, and long-term implications (9, 10). The term ECC intrinsically describes a disease process confined to the primary dentition, a period conclusively ended by the emergence of the first permanent tooth. The caries susceptibility of a newly erupted permanent tooth is uniquely high. These teeth undergo a prolonged post-eruptive maturation phase, where initially porous and poorly mineralized enamel requires up to two years or more to become resistant to acid dissolution (9). During this critical window, the immature permanent enamel is often more susceptible to rapid caries progression than the older primary enamel it replaces (10). A lesion in a first permanent molar is, thus, a disease of an immature permanent dentition, a distinct entity from caries in long-exposed primary teeth.

The site and aetiology of the disease also reflect a different risk paradigm. While ECC is strongly associated with specific infant feeding behaviours leading to characteristic lesions on maxillary incisors (11), caries in the early mixed dentition primarily affects the pit and fissure system of the first permanent molar. This aetiology is tied to dietary habits, oral hygiene, and morphological vulnerability as the child gains dietary independence, not nocturnal feeding (12). Grouping a smooth-surface lesion on a primary incisor with an occlusal lesion on a permanent molar under one diagnostic category obscures these critical etiological differences.

In addition, the long-term prognostic significance is fundamentally different. Although ECC in primary teeth is a predictor of future caries, the disease in a primary tooth is transient (13). In contrast, caries in a permanent tooth has lifelong consequences; the restoration or loss of a first permanent molar, a keystone in the developing occlusion, constitutes a permanent impact on oral health and function (14). Classifying this lifelong disease event under a category designed for a transitional dentition and diagnosing caries in permanent teeth as ECC is an anachronism that fails to acknowledge the biological milestone of permanent tooth eruption, and misunderstands its clinical gravity, leading to a blurred aetiological understanding and imprecise clinical management.

## Fostering integration with general child health and development

Beyond biological precision, a revised definition is essential for integrating oral health into the broader ecosystem of child health. The medical field utilises standardised age groupings that are intrinsically linked to well-established developmental trajectories, guiding everything from well-child visits and vaccination schedules to developmental screening. Within this paradigm, early childhood is defined as encompassing the period from birth to 48 or 60 months, a phase characterised by rapid brain growth, the acquisition of foundational motor and language skills, and the formation of primary attachment relationships (15). A child beyond 60 months is universally classified in paediatrics as a preschooler, a distinct developmental stage marked by advancing social-emotional competencies, more complex cognitive abilities, and the transition toward the structured learning environment of formal schooling (16).

The current dental definition of ECC, extending to 71 months, creates a disruptive disconnect with these established medical and developmental classifications (17), resulting from conceptual misalignment. When a paediatrician assesses a child over 60 months old, their clinical approach is informed by the preschooler developmental paradigm, which includes anticipatory guidance on topics like peer interactions, pre-literacy skills, and nutritional independence. Forcibly categorising this same child's oral health status within an early childhood context ignores the developmental shift the child has undergone and creates a cognitive dissonance for the healthcare provider. The discordance hinders the seamless integration of oral health risk assessments into the well-child visit, as the paediatrician's developmental framework for a preschooler may not automatically engage the specific preventive counselling protocols, such as discussing the cessation of non-nutritive sucking habits or the management of on-demand snacking, that are paramount for the toddler in the true early childhood period (18). Integrating oral health into standardised paediatric well-child visits is more feasible when age categories align, and paediatricians and dentists can collaborate using shared developmental frameworks, facilitating referrals, coordinated counselling, and integrated electronic health record prompts that reflect consistent age-based risk assessments.

This misalignment has consequences for both clinical care and public health. It complicates interdisciplinary communication and the development of unified electronic health record prompts, as a single patient is simultaneously classified in two different developmental categories across medical and dental domains (19). This inconsistency directly impedes the creation of cohesive, life-course health metrics that track a child's holistic well-being. As global initiatives, silos between oral and systemic health need to be dissolved, and aligning our fundamental definitions is a necessary foundational step to achieving this (20). Harmonising the ECC age limit with common paediatric age brackets is a commitment to a bio-psycho-social model of care for the child. It ensures that oral health is viewed and addressed as an integral component of the child's continuous developmental journey, thereby streamlining data collection, fostering collaborative research, and supporting a more holistic and effective approach to ensuring every child's well-being.

By framing ECC as a condition of early childhood, it captures the patient-centered reality of caries as a chronic, multifactorial disease process that begins long before cavities form. The shift from a tooth-centric perception of ECC can facilitate health education opportunities that parallel other early childhood interventions, such as immunisations and developmental screenings. These interventions can serve as opportunities for regular dental visits and routine parental counselling on oral hygiene and diet for oral health (21). Harmonising the ECC definition with paediatric age brackets allows for better integration of oral health into well-child visits. Paediatricians can incorporate caries risk assessments and fluoride varnish applications into routine care, ensuring that oral health is addressed alongside other developmental priorities (18, 22).

## Sharpening public health focus: the first 1,000 days and beyond

Furthermore, adopting a definition that concludes at 60 months provides a strategic public health reframing that creates a clear conceptual boundary that directs primary attention and resources upstream to the most critical, evidence-based preventive window: the first 1,000 days of life, spanning from conception to a child's second birthday. This period represents a unique phase of developmental programming and plasticity where the oral microbiome is established, foundational dietary preferences are shaped, and family-based preventive care routines are initiated (23, 24). By defining ECC as a condition of early childhood that culminates at age 60 months, we strategically underscore that the disease process is seeded in this earliest period. The current definition, extending to 71 months, risks diluting this vital message by implicitly spreading the at-risk period across six years, thereby blurring the unparalleled urgency of intervention during the first 1,000 days, a period recognised as the most receptive window for establishing lifelong health trajectories (25).

Evolving evidence underscores that the events of this window are paramount in setting the trajectory for ECC (26). Dietary habits forged during the first 1,000 days are especially consequential. The transition from a liquid diet to the introduction of solid foods is a period of significant risk. The type, frequency, and cariogenicity of complementary foods introduced can either protect or predispose the child to disease. Frequent exposure to sugary snacks and drinks, often driven by marketing and parental misconceptions about weaning foods, can rapidly accelerate the caries process in susceptible, newly erupted enamel (27).

The relationship between breastfeeding and caries risk exemplifies the multifactorial nature of ECC and must be contextualised within the broader model of interacting determinants. While exclusive breastfeeding for the first six months is universally recommended for its systemic benefits, research indicates that prolonged, on-demand breastfeeding beyond 12 months of age, particularly at night without subsequent oral hygiene, can be a significant risk factor for ECC (28, 29). Furthermore, this risk intersects with socio-environmental determinants, such as maternal education (30), access to preventive dental care (31), and familial oral health literacy (32–34), which influence the adoption of protective behaviours like early dental visits and fluoride use. This highlights the complex interplay between a beneficial practice and oral disease risk, emphasising the need for targeted, non-stigmatising counselling that addresses the entire caregiving context during this specific developmental period, rather than focusing on any single behaviour in isolation.

Lowering the age limit to 60 months performs a crucial signalling function for health systems and caregivers alike. It reinforces that the foundation for a caries-free primary dentition is laid in infancy, and recognises that the goal is to promote the overall health and development of the child, for whom untreated ECC has profound consequences, including pain, infection, impaired growth (35) and brain development (36), and diminished quality of life (37). Public health messaging, clinical guidelines, and resource allocation can thus be targeted with greater precision to the stages of greatest biological vulnerability and maximal caregiver influence, predominantly within those first 1,000 days (38). It also powerfully reinforces the life-course paradigm that prevention must be prioritised in infancy and toddlerhood (39). This alignment ensures that educational efforts for parents and training for primary care providers emphasize prenatal counselling, early dietary habits, timely fluoride use, and the first dental visit, all anchored within this golden window where they can have the greatest impact, creating a clearer demarcation for evaluating early interventions.

## Enhancing epidemiological precision through stratification

From a research perspective, precision in age stratification is paramount. The period from birth to five years encompasses rapid and distinct developmental milestones. Aetiologically, ECC typically follows a classic pattern in infants and toddlers under three years, driven by feeding practices and closely tied to maternal and infant microbiology and feeding behaviours. Key determinants include the timing of *Streptococcus mutans* transmission from mother to child, frequent consumption of sugar-sweetened liquids from a bottle, and breastfeeding practices, particularly nocturnal feeding (26, 28). Prolonged exposure to fermentable carbohydrates from bottles or at-will breastfeeding leads to acid pooling around the maxillary incisors, while the mandibular incisors are protected by the tongue and sublingual saliva (3). This reflects a behavioural aetiology centered on caregiver-dependent nutrition and oral hygiene.

In contrast, by 61 to 71 months, caries patterns shift significantly due to dietary independence, involving frequent snacking on cariogenic foods, the consumption of sugar-sweetened beverages, the adequacy of parental supervision of brushing, as well as the child's own emerging behavioural autonomy, including oral hygiene habits (11). At this stage, children increasingly exert influence over their own dietary choices and oral hygiene routines, marking a transition from caregiver-mediated to child-driven health behaviours. Dietary exposure becomes less controlled by parents, with frequent snacking on cariogenic foods and consumption of sugar-sweetened beverages becoming common. Simultaneously, oral hygiene practices depend increasingly on the child's own manual dexterity, motivation, and parental supervision rather than direct caregiver (40, 41). This behavioural shift modifies the caries risk profile, moving it away from the infant-feeding-dependent model characteristic of early childhood and toward a pattern influenced by school environment, peer behaviours, and growing personal agency (25). In addition, lesions increasingly occur on the proximal surfaces of primary molars and in the deep pits and fissures of newly erupted permanent molars, aligning with dietary habits, emerging oral hygiene practices, and tooth morphology rather than infant feeding behaviours (10). Conflating these etiologically distinct presentations under a single diagnostic category obscures trends in surveillance, complicates the evaluation of age-specific interventions, and ignores the evolving nature of the cariogenic challenge as a child develops.

Clinically, lowering the ECC age limit to 60 months allows for meaningful adjustment that modernises evidence-based guidelines, all contributing to more effective, individualised care for children. For example, preventive guidance relevant to a one-year-old, such as avoiding bedtime bottles, bears little relevance to a five-year-old, who may require strategies focused on snacking management, reinforcement of supervised brushing, and timely application of pit-and-fissure sealants on newly erupted permanent molars. This shift ensures that anticipatory guidance is developmentally congruent, enhancing parental engagement and adherence. The diagnostic inconsistency of labelling caries in early-erupted permanent teeth as ECC is highlighted in clinical guidelines that recommend distinct management pathways for caries in primary vs. permanent dentitions (42, 43). Furthermore, studies show that restorative outcomes and long-term oral health impacts differ significantly between the two (44, 45). These underscore the need for clear diagnostic boundaries. A refined ECC definition would reduce diagnostic ambiguity, helping clinicians apply these guidelines more consistently and avoid misclassifying early permanent tooth caries as ECC, which could lead to under-treatment or inappropriate monitoring.

Finally, redefining ECC would catalyse the updating of risk-based clinical guidelines. Current caries risk assessment tools, such as the Caries-risk Assessment Tool from the American Academy of Pediatric Dentistry, include age parameters that may not reflect modern eruption patterns (46). A revised ECC boundary would prompt a re-evaluation of these tools to better distinguish between risks in the exclusive primary dentition phase and those in the early mixed dentition. This could lead to more tailored risk categories, refined recall schedules, and personalised preventive prescriptions. Furthermore, it would encourage the development of transitional guidelines for children around age 5, who are at the interface of early childhood and school-age dental health priorities.

While some may argue that maintaining a broader age range simplifies global reporting, the biological and clinical evidence support a more nuanced approach. The proposed change does not complicate reporting; rather, it refines it. Standardised age strata (0–4 years for ECC, 5–12 years for school-age caries) are already familiar in public health. Transitional guidelines can support implementation in diverse settings. Refining the ECC definition to culminate at age 60 months would create a more homogeneous cohort, truly representing the period of exclusive primary dentition. This would allow for a cleaner comparison with the subsequent school-age cohort and enable researchers to ask more precise questions, thereby strengthening the validity of analytical models and ensuring that public health resources and clinical guidance are deployed with maximum impact at the most vulnerable and appropriate stages (47).

Lowering the upper limit to 60 months creates a more homogeneous cohort representing the period of exclusively primary dentition. This allows for a cleaner comparison with the subsequent school-age cohort (ages 5–12), which is characterised by key developmental and dental milestones, beginning with the transition from the preoperational stage to the concrete operational stage of cognitive development (48), into formal schooling, and ending at the onset of adolescence (49). Thus, age stratification needs to correspond to the sensorimotor and preoperational stages (the period from birth to 60 months), during which learning, behavior, and health habits are heavily mediated by caregivers. Such clarity is essential for accurately tracking trends and evaluating the long-term impact of early preventive programs. It also enables researchers to ask more precise questions, thereby strengthening the validity of analytical models and public health decisions.

## Implications for public health and health system integration

Adopting a refined ECC definition concluding at 60 months is a step towards enhancing public health infrastructure and integrating oral health into holistic child healthcare that can catalyse the building of a more unified, equitable, and developmentally attuned system of child healthcare. The multifaceted benefits of this redefinition include:

(a) **Strengthening Surveillance and Data Equity**: A more precise ECC definition would improve epidemiological surveillance by creating a homogeneous cohort truly representative of the exclusive primary dentition period. This allows for tracking of disease trends and clearer comparisons across age groups. Such clarity is essential for evaluating interventions and monitoring disparities effectively (50, 51). Importantly, a refined definition enhances data equity by enabling early identification of high-risk and underserved populations. With clearer age stratification, public health systems can better detect disparities, direct resources where they are most needed, and monitor the impact of targeted programs aimed at reducing oral health inequalities.
(b) **Aligning with Paediatric Well-Child Frameworks**: Harmonising the ECC age limit with established paediatric age classifications ensures that oral health guidance is delivered in a developmentally relevant context. This alignment facilitates the integration of caries risk assessments, fluoride varnish applications, and preventive counselling into routine well-child visits (15, 18). By synchronising dental and medical developmental frameworks, healthcare providers can offer timely, stage-appropriate interventions that improve parental engagement, adherence, and overall health outcomes.
(c) **Enabling Interdisciplinary Collaboration**: A unified age classification mitigates inconsistencies in electronic health records and clinical categorisation that often fragment care between medical and dental providers. This coherence supports shared decision-making, integrated care pathways, and efficient resource use, embodying a team-based approach to child health (19, 52). It also simplifies communication across disciplines, enabling paediatricians and dentists to collaborate effectively using a common developmental language.
(d) **Informing Targeted Intervention Planning, Policy Integration and Advocacy**: From a programmatic perspective, a sharper definition refocuses public health efforts on the most vulnerable and influential period. It allows for the precise targeting of resources and messages to establish healthy foundations, emphasising prenatal care, early dietary habits, and timely first dental visits. This supports a proactive, life-course model of prevention over restorative care (33, 53). By concentrating efforts on the period of greatest biological vulnerability and behavioural influence, public health programs can shift from a reactive, restorative model to a proactive, preventive life-course approach.

This definitional refinement operationalises global calls to integrate oral health into primary healthcare and universal health coverage. It enables the inclusion of oral health metrics in national child health surveillance systems, supports policy coherence across health and education sectors, and strengthens advocacy to prioritise ECC within broader child development agendas (19, 20). A clear, biologically aligned definition provides a stronger evidence base for policymaking, funding allocation, and cross-sectoral initiatives aimed at improving child health outcomes.

The successful adoption of this refined definition will require a coordinated, phased implementation strategy sensitive to diverse global contexts. In settings where precise age verification is challenging, clear operational guidelines, such as leveraging birth registries or integrating oral health assessments into existing maternal and child health milestones, can facilitate accurate classification. This transition must be supported by interoperable electronic health records capable of tracking oral and systemic health metrics seamlessly, moving from a tooth-focused model to a whole-child care framework. Ultimately, redefining ECC is a catalyst for building more unified, equitable, and developmentally attuned systems of child healthcare.

## Conclusion

The proposal to lower the upper age limit for ECC from 71 months to 60 months is a forward-looking adjustment grounded in biological reality, methodological rigor, and a commitment to integrated healthcare. It represents a shift in paradigm from a reactive, tooth-counting model that spans both infancy and the preschool years to a proactive, health-promoting model that prioritizes prevention at its source. It ensures that the formidable public health challenge of ECC is met with interventions that are developmentally appropriate and temporally precise, empowering families and healthcare systems to act when their impact can be greatest. The change also aligns dental terminology with paediatric developmental stages, including the recognised transition toward behavioural autonomy after age five, thereby breaking down long-standing barriers to holistic childcare. By adopting this refined definition, the dental community can enhance the translation of its research to clinical practice and the relevance for integrated clinical guidelines for childcare, and its capacity to partner effectively in promoting the comprehensive health of all children during their early development.
